# A Simple Diagnosis-Related Groups-Based Reimbursement System Is Cost Ineffective for Elderly Patients With Displaced Femoral Neck Fracture Undergoing Hemiarthroplasty in Beijing

**DOI:** 10.3389/fmed.2021.733206

**Published:** 2021-12-15

**Authors:** Hui-ming Peng, Yuan Xu, Pu-wo Ci, Jia Zhang, Bao-zhong Zhang, Xi-sheng Weng

**Affiliations:** ^1^Department of Orthopaedics, Peking Union Medical College Hospital, Chinese Academy of Medical Science, Peking Union Medical College, Beijing, China; ^2^Department of Thoracic Surgery, Peking Union Medical College Hospital, Chinese Academy of Medical Science, Peking Union Medical College, Beijing, China; ^3^Department of Medical Record, Peking Union Medical College Hospital, Chinese Academy of Medical Science, Peking Union Medical College, Beijing, China

**Keywords:** DRG-based reimbursement system, hemiarthroplasty, femoral neck fracture, orthopedics, surgery

## Abstract

Displaced femoral neck fractures (FNF) in the elderly are a major public health concern that necessitates hemiarthroplasty (HA) as the mainstay treatment option. Diagnosis-Related Groups (DRG) are a patient classification system that categorizes patients based on the resources expended on them. The first objective of this study was to evaluate if a simplified DRG-based reimbursement system in Beijing would lower total HA treatment costs for elderly patients with displaced FNF. In addition, we aimed to determine how age, gender, year of admission, length of in-hospital stay, and the Charlson index affected total treatment costs. This retrospective study included 513 patients from the Peking Union Medical College Hospital. The patients were diagnosed with unilateral displaced femoral neck fractures and had HA. Medical information was gathered, including baseline demographic and clinical data, as well as treatment costs. Patients were classified into two groups: those who spent more than the predetermined cut-off cost and those who did not. The cost did not include the use of a bipolar prosthesis. Data from the two groups were compared, and multiple regression analysis models were constructed. The median total cost of treatment was ¥49,626 ($7,316). The majority of the patients (89.7%; 460/513) were categorized as exceeding the cost cut-off. Multiple linear regression analysis revealed that total treatment cost was positively correlated with age (*p* < 0.01) and the duration of in-hospital stay (*p* < 0.01) but not with gender (*p* = 0.160) or the Charlson index (*p* = 0.548). On implementing the DRG-based reimbursement system, the overall treatment costs increased by ¥21,028 ($3,099) (*p* < 0.01). The implementation of simplified DRG-prospective payment systems did not result in a significant reduction in total treatment costs for elderly patients with FNF who underwent HA in Beijing. The overall cost of treatment was associated with several factors, including age, length of hospitalization, and year of admission.

## Introduction

Osteoporosis is on the rise around the world, resulting in an increasing number of hip fractures, which are associated with a high rate of morbidity and death. Additionally, the costs of corrective surgeries to treat fractures put a significant burden on healthcare budgets (1). Femoral neck fractures (FNFs) make up about 57% of all reported hip fractures (2). Internal fixation and closed or open reduction are two treatment options for FNFs. Hip hemiarthroplasty (HA) and total hip arthroplasty (THA) are also options (THA). Internal fixation for older individuals with displaced fractures increases the likelihood of non-union. Studies have demonstrated that HA improves hip function, quality of life, and reduces hip-related complications and reoperations (3). THA, on the other hand, is considered to be a more expensive treatment. For these reasons, HA has remained the mainstay treatment option for elderly patients with these fractures in Beijing, China. The healthcare system must focus on improving patient outcomes and reducing the cost burden of patients with FNFs who seek HA.

Diagnosis-Related Groups (DRGs) are Patient Classification Systems (PCSs) that are commonly used to classify, benchmark, and ultimately pay for hospital care (4). According to clinical data (such as diagnoses and procedures), demographic data (such as age and gender), and resource consumption data (such as hospitalizations), cases are categorized (including costs, duration of hospitalization). DRGS was originally designed as a management tool for clinicians to monitor service quality and utilization, but it is now used as a prospective payment system in many countries (5). In 2011, Beijing, China implemented a DRG-based reimbursement system for the treatment of FNFs. Beijing's FNF expenditures were limited under this arrangement to a fixed payment of $3,573, which included the cost of the prosthesis, at $8,100. Therefore, if the patient's actual medical expenses, excluding the costs of the prosthesis, exceed 16, 145 ($2,379), the hospital can only charge the patient 16,145. This is the difference between the declared total costs and those for the prosthesis. If the patient spends less than this amount, the system will cover the actual cost (6). Reducing overall treatment costs was the original goal of the DRG reimbursement system. Concerns about the complexity and diversity of patient situations and needs, which could lead to increased postoperative difficulties and treatment costs, led to some opposition to the system's introduction. The use of THA for FNF was found to be associated with higher rates of complications, the need for post-discharge inpatient care, and higher rates of unplanned readmission in a US study conducted between 2008 and 2016 by Charette et al., compared with OA, suggesting a need to modify healthcare reimbursement policy (7). To the best of our knowledge, no data or research exists in China demonstrating the efficacy of the DRG-based approach. Because of this, it is not yet clear if this method can meet the needs of older patients who are undergoing HA for the treatment of FNF.

A total of 513 patients with FNF who underwent HA at the Peking Union Medical College Hospital between January 1, 2006, and June 31, 2017, were studied in this retrospective analysis. After establishing the DRG-based reimbursement system for FNF patients, we predicted that the total cost of treating HA would greatly surpass the assigned fixed rates for FNF patients. A multiple regression model was used to examine the risk factors associated with exceeding the allocated treatment expenditures in this study.

## Materials and Methods

A retrospective review of the data of patients who underwent HA for FNF at our hospital between January 1st, 2006, and June 30th, 2017 was conducted. The Peking Union Medical College Hospital's Institutional Review Board approved the study protocol. Based on the following information, patient records had to meet the inclusion criteria: (1) a patient aged 70 or older; (2) an X-ray-confirmed unilaterally displaced femoral neck fracture (ICD-10: S72.00); and (3) hemiarthroplasty as a treatment option (ICD-9-CM-3: 81.51-81). Records were excluded if the patient (1) had other surgeries, such as total hip arthroplasty (THA) or revision hip arthroplasty; (2) was enrolled in a clinical trial (such patients typically receive a fee waiver on treatment costs); and (3) the patient's costs were not covered by this reimbursement system. All data, including demographics, treatment-related costs, comorbidities, and length of hospital stay, were obtained from the hospital's patient electronic medical record system. The overall costs composed of medication (all drugs, including coagulation factor products, albumin, and immunoglobulin products), examination (pathological, laboratory, and other clinical diagnostic procedures), treatment procedures (non-operative treatment, including nursing care, rehabilitation management, and physical therapy) and surgery (including bipolar prosthesis). The Charlson Comorbidity Index was calculated using medical records to assess the patients' physical health status (8).

### Statistical Analyses

Data were collected in Excel (version 2016, Microsoft Corporation, Inc., Redmond, WA, USA) and analyzed in SPSS (version 22.0, SPSS Inc., Chicago, IL, USA). To examine inter-group differences, the Mann-Whitney *U*-test was used. To predict total treatment costs, a multiple linear regression model was constructed. The statistical significance level was set at *p* < 0.05.

## Results

### Patient Characteristics

A total of 513 patients with displaced FNF who met the study inclusion criteria were enrolled in the study. Table 1 summarizes baseline information and demographic data. The patients' mean age was 79.48.6 years, with the majority of them being female (*n* = 365, 71.2 %). The average length of stay in the hospital was 18 [IQR = 13] days. Following the implementation of the DRG-based reimbursement system on January 1, 2011, 348 patients were admitted, accounting for 67.8% of all patients. For all patients, the Charlson index was a median of 1 [IQR = 2].

**Table 1:** Descriptive statistics for the study population.

**Demographic data**	***N* = 513**
Age (years)	79.4 ± 8.6
**Gender**	
Female	365 (71.2%)
Male	148 (28.8%)
**Year of admission**	
2006-2010	166 (32.3%)
2011-2017	348 (67.7%)
Length of in-hospital stay (days) (Median, IQR)	18 (13)
Charlson index (Median, IQR)	1 (2)
**Costs (RMB;** ¥**) (Median, IQR, %)**	
Total	49,626 (23,433) (100%)
Drug	7,934 (9,026) (16.0%)
Examination	6,600 (6,578) (13.3%)
Treatment	2,407 (3,472) (4.8%)
Surgery	28,920 (31,466) (58.3%)
Other	3,765 (1,622) (7.6%)

*IQR, Interquartile range; RMB, Renminbi, $1≈¥6.8*.

### Cost Assessment and Comparison

Overall, the median total cost of hospitalization was $49,626 [IQR = 23,360] ($6,789; IQR = $3 196), with surgical costs exceeding other treatment-related costs (28,920, 58.3%). Medication (7,934, 15.9%) and medical examination costs (6,600, 13.3%) accounted for a significant portion of the costs, while treatment accounted for only 4.9% cost (2,407).

Patients with costs that exceeded 16,145 (calculated by subtracting the allocated costs of prosthesis use (8,100) from the overall assigned cost of care of FNF (24,245) according to the simplified DRG-based system implemented in Beijing, China) were classified as more costly, while those with medical bills that were <16,145 were classified as less costly. The majority of patients (460, or 89.7%) were classified as more expensive. There were no significant between-group differences in age (79.4 years vs. 79.3 years, *p* = 0.723) or gender (*p* = 0.585) (Table 2). However, the more expensive group had a longer in-hospital stay than the less expensive group (19 days vs. 9 days, *p* < 0.01). Following the implementation of the new reimbursement system, the majority of patients in the more expensive category (63.9%) were admitted. In comparison, all patients in the less costly category received their admission after the implementation of the DRG-based system (*p* < 0.01). The Charlson index was higher in the more expensive group than in the less expensive group (1 vs. 0, *p* = 0.02).

**Table 2:** Data for patients in the more costly and less costly groups.

**Patient characteristic**	**More costly**	**Less costly**	***P***
*N*	460	53	
Mean age (years)	79.4 ± 8.6	79.3 ± 8.0	0.723
Gender (male)	131 (28.5%)	17 (32.1%)	0.585
Duration of hospital days (median, IQR)	19 (11)	9 (4.5)	<0.01
Year of admission (2011-2017)	294 (63.9%)	53 (100.0%)	<0.01
Charlson index (median, IQR)	1 (2)	0 (1)	0.02

*P, Mann-Whitney U-test*.

### Multiple Regression Analysis Model

We developed a multiple regression analysis model to predict total costs and explore the influence of gender, age, year of admission, length of in-hospital stay, and the Charlson index on costs (Table 3). In line with the previous findings, total costs were not correlated with gender (*p* = 0.160) or the Charlson index (*p* = 0.548). However, there was a positive correlation (*p* < 0.01) between the length of in-hospital stay and total costs. After adjusting for gender, year of admission, and the Charlson index, total costs increased by $4,104.1 per day. The year of admission also had a significant impact on total costs (*p* < 0.01). Total costs for patients admitted since 2011 were 21,028 higher than for patients admitted before 2011. In this model, age was a significant influencing factor (*p* < 0.01), with the total cost increasing by 770.1 for every year after the age of 70.

**Table 3:** Multiple regression analysis model of total treatment costs.

**Model parameter**	**β**	***P*-value**
(Constant)	−121892.9	<0.01
Gender	7505.8	0.160
Age	770.1	<0.01
Year of admission	21027.9	<0.01
Length of in-hospital stay	4104.1	<0.01
Charlson index	1233.1	0.548

*R^2^, 0.92; adjusted R^2^, 0.862; standard error of the estimate, 54317.9; ANOVA: F, 638.1*.

*Gender: 0, female and 1, male; Year of admission, 0; 2016-2010, 1; 2011-2017*.

## Discussion

The current study found that simplifying the DRG-PPS did not significantly reduce the total cost of HA for patients with FNF. In fact, after implementing the above-mentioned cost-estimating system, the total treatment cost per patient increased by $8,670. After adjusting for age, gender, length of in-hospital stay, and the Charlson index, the total cost per patient increased by up to $21,028 ($3,094) (*p* < 0.01). Notably, surgical-related expenditure (7,937) was the largest component of the overall cost, owing primarily to the cost of using a prosthesis. Because the cost of prosthesis use was not calculated in the reimbursement system, the findings suggest that medical institutions may have chosen more expensive prostheses after the DRG-PPS was implemented in 2011. Other factors, such as price adjustments, economic growth, and increased attention to healthcare, could explain the observed cost increase. Notably, all patients in the less expensive category were admitted after 2011, indicating that the DRG-PPS had a positive effect on treatment costs. This finding could be attributed largely to the observed decrease in examination costs after 2011 (1,628).

The predominant healthcare payment system in most Chinese hospitals is fee-for-services. Hospitals are paid based on the medical services they provide under this system. As a result, hospitals frequently over-treat in order to maximize profits. Reforms in healthcare payment policy are required to reverse this trend. In light of this, the DRG-based payment system, which is thought to be superior in terms of cost containment and efficiency, has received increased attention. Several European countries have implemented DRGs or similar grouping systems as instruments for hospital reimbursement over the last 30 years. In the 1990s, China began implementing DRG-based prospective payment systems (DRG-PPSs). Jiangsu, a Chinese province, piloted a reimbursement system reform in 2001. The central government promoted the resulting DRG-based system in seven additional provinces in 2004 (out of a total of 34 provinces). Other provinces, including Beijing, adopted the system in 2009. Unlike the effective and well-executed models in developed countries, however, the system adopted in China was a prototype known as the simplified DRG-PPS, also known as the “ceiling price for a single disease” (6). In Beijing, FNF was formally incorporated into the system in 2011. The Beijing Medical Insurance Affairs Management Center paid hospitals fixed amounts for services such as prostheses and medical services during hospitalization under this system, which was based on the previous three-year institutional average cost.

Liu et al. reviewed 22 Chinese studies on the use of DGR-PPS in 12 provinces. According to the findings of the study, simplified DRGs are useful in controlling hospitalization costs (6). However, in the article, studies were chosen from either Chinese literature or Chinese government reports, resulting in a lack of high-quality research. In reality, the system's implementation was contentious in practice. In comparison to systems used in developed countries, China's reimbursement system was extremely simplified. The system, for example, only covered a few simple and common diseases, such as simple appendicitis. Second, this simplified DRG-PPS only takes into account primary diagnoses and ignores other patient characteristics such as age, gender, disease severity, and health- or disease-related complications) (6). According to Mathauer and colleagues' findings, deficiencies in coding standardization, data availability, and information technology make the scientific implementation of DRGs difficult in low- and middle-income countries (9). Furthermore, the criteria for determining cost limits were primarily based on historical costs, which could be inaccurate or out of date (10).

FNF is currently treated using a variety of approaches, including open reduction and internal fixation, HA, THA, and conservative therapies. Arthroplasty is becoming more common in Northern Europe (11) and the United States (12), with HA being a more popular option for elderly patients than THA. THA, on the other hand, has been linked to better outcomes. According to Vos et al., when compared to traditional THA, HA is associated with more inpatients experiencing adverse events and a longer length of in-hospital stay (13). Althausen et al. also noted that patients who underwent HA due to FNF had significant comorbidities, which resulted in higher costs and longer hospital stays, further increasing treatment costs (14). Only patients who had undergone HA were enrolled in this study. Because HA was suitable for patients with shorter life expectancies or lower functional needs, the subjects in our study were older or had more complications than typical FNF patients. These study subjects' characteristics may also have resulted in higher total costs. Previous research has suggested that the initial inpatient cost for HA is higher than for internal fixation (15).

Multiple regression analysis revealed that age had a significant impact on treatment costs (*p* < 0.01). After adjusting for gender, year of admission, duration of in-hospital stay, and the Charlson index, the total cost increased by 770 for each additional year. This finding is consistent with the findings of similar studies among hip fracture patients, which found that older patients had slightly higher costs (16). Notably, patients undergoing HA in those studies were typically older than 70 years old, whereas, in our study, the average age of patients was 79.48.6 years. The length of hospital stay was also significantly associated with total cost (*p* < 0.01), with costs increasing by $4,104 per day. The current study's median length of in-hospital stay was 18 days, which was slightly longer than in previous studies. In a study conducted in the United States, Castelli reported an average length of stay of 21 days, with a median of 15 days, among patients with femur neck fractures who were 81 years old on average (16). The slightly longer hospital stay observed in our study could be attributed to more disease-related complications among our study participants, as well as a lack of a referral system, both of which could have resulted in higher total costs.

To assess a patient's health status, several scoring systems have been developed, including the American Society of Anesthesiologists (ASA) score, the Elixhauser score, and the Charlson index (17). The Charlson Comorbidity Index is the most widely used of the available and validated comorbidity measures (18). Johnson et al. (19) discovered a positive relationship between the Charlson index and total cost, which was not found in the current study (*p* = 0.548). We hypothesized that one of the reasons for this disparity is that the Charlson index focuses on organ functions rather than fracture-related complications. Peptic ulcer disease, fluid and electrolyte disorders, psychoses, and coagulopathy, as other studies have shown, are important determinants of treatment-related costs (17); however, none of these conditions are assessed in the Charlson index. This could explain why we didn't find a link between the Charlson index and the length of in-hospital stay (*p* = 0.04, Supplementary Table S1).

Although the current study provides us with useful insights, we recommend that we interpret the findings with caution due to some limitations. Firstly, the study site is a government-designated referral medical center for undiagnosed and acute diseases. As a result, patients with comorbidities were more willing to be hospitalized, which may have resulted in an increase in treatment costs. Second, our study drew on data collected over 11 years. As previously stated, price adjustments, economic growth, and increased focus on healthcare should all be considered. Third, because this was a retrospective study, the Charlson index was calculated using the patients' medical records, which may have resulted in the underestimation of the scores. Furthermore, age is implicitly considered in the Charlson index, but no significant collinearity was observed in the multiple regression analysis model; a finding that suggests the use of alternative scoring systems. Charslon index is debated however still it was the most comprehensive and appropriate index available for our study.

According to our findings, the simplified DRG-PPS did not significantly reduce the total cost of HA for elderly patients with FNF. The overall cost was influenced by several factors, including age, length of in-hospital stay, and year of admission. To reduce total costs associated with FNF treatment, we recommend that cost estimates be based on empirical evidence and that a reasonable payment standard be developed for various patients, particularly those with disease-related complications and comorbidities. Furthermore, we recommend that the reasons for in-hospital stay extensions be evaluated in order to reduce any invalid hospital stays.

## Data Availability Statement

The original contributions presented in the study are included in the article/Supplementary Material, further inquiries can be directed to the corresponding author/s.

## Ethics Statement

The studies involving human participants were reviewed and approved by Peking Union Medical College Hospital. The patients/participants provided their written informed consent to participate in this study.

## Author Contributions

H-mP and YX designed and wrote the manuscript. P-wC and JZ collected the data. B-zZ and X-sW analyzed the data. All authors approved the manuscript.

## Conflict of Interest

The authors declare that the research was conducted in the absence of any commercial or financial relationships that could be construed as a potential conflict of interest.

## Publisher's Note

All claims expressed in this article are solely those of the authors and do not necessarily represent those of their affiliated organizations, or those of the publisher, the editors and the reviewers. Any product that may be evaluated in this article, or claim that may be made by its manufacturer, is not guaranteed or endorsed by the publisher.
