# Supplementary material for: A Simple Diagnosis-Related Groups-Based Reimbursement System Is Cost Ineffective for Elderly Patients With Displaced Femoral Neck Fracture Undergoing Hemiarthroplasty in Beijing
Source: Front Med (Lausanne). 2021 Dec 15;8:733206. doi: 10.3389/fmed.2021.733206 (PMC8715944; doi:10.3389/fmed.2021.733206)
Supplement: Supplementary file 1 [file Table_1.DOCX]

**Table S1. Comparison of median costs before and after 2011**

| **Item** | **2006-2010 (RMB)** | **2011-2017 (RMB)** |
| --- | --- | --- |
| Drug | 7867 | 8006 |
| Examination | 8103 | 6475 |
| Treatment | 1197 | 3044 |
| Surgery | 23128 | 31065 |
| Prosthesis | 22248 | 28721 |
| Other | 3167 | 3500 |
| Total | 43462 | 52132 |
